# Public health round-up

**DOI:** 10.2471/BLT.17.010217

**Published:** 2017-02-01

**Encouraging people to seek help for depression**

World Health Day this year on 7 April aims to make people more aware of depression and the fact that it can be prevented and treated. This is one of the posters for the “Depression: let’s talk” campaign, illustrating that talking is often the first step towards getting better.

For more information: http://www.who.int/depression

**Figure:**
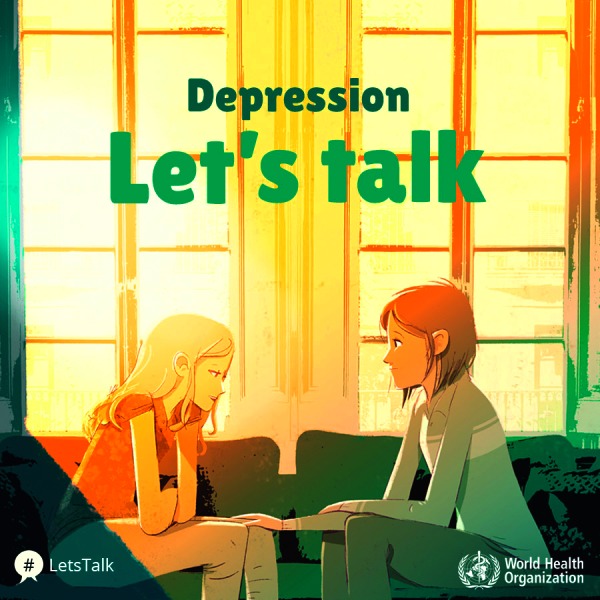
WHO

## Measles vaccination in Nigeria

A measles vaccination campaign was launched last month in north eastern Nigeria to protect more than four million children against the highly contagious and sometimes fatal disease.

Children between the ages of six months and 10 years were vaccinated in Borno, Yobe and Adamawa, Nigerian states where the health services have been disrupted for more than four years because of the conflict in Borno.

The campaign overcame major logistical challenges of insecurity, difficult terrain, poorly functioning health facilities and the need for cold chain conditions. 

The humanitarian crisis caused by the conflict in Borno State has resulted in more than 1.4 million displaced people in more than 100 camps.

"Many of the children targeted for measles vaccination have severe malnutrition, making them extremely vulnerable to serious complications and death from measles," says Dr Wondimagegnehu Alemu, World Health Organization (WHO) Representative in Nigeria.

The measles campaign was planned using population data gathered by Nigeria’s polio vaccination programme and with help from its staff who have immunization experience across the country.

During the measles campaign, the vaccine was given to children between the ages of one and 10 years, along with deworming medication and vitamin A supplements to the children who were aged between six months and five years.

To prevent duplication, especially in schools and camps for displaced people, vaccination cards were issued to all vaccinated children and their thumbs were marked with pens.

From early September to 18 December 2016, an early warning, alert and response system established by WHO reported more than 1500 suspected measles cases in Borno State alone.

WHO is supporting the health authorities in the three Nigerian states by providing expertise in logistics, data management, training, monitoring and evaluation, human resources and waste management.

Other partners involved in the campaign include the United Nations Children’s Fund, the United States Centers for Disease Prevention and Control (CDC) and nongovernmental organizations including Médecins Sans Frontières (MSF).

http://www.who.int/features/2017/measles-vaccination-nigeria

## Trial finds Ebola vaccine effective

A two-year clinical trial involving 11 841 people in Guinea during 2015 found an experimental vaccine to be highly protective against Ebola virus disease, according to the results published late last year in the *Lancet*.

Among the 5837 people who received the vaccine, called rVSV-ZEBOV, no Ebola cases were recorded 10 days or more after vaccination. In comparison, there were 23 cases among those who did not receive the vaccine during the same time period.

The trial was led by WHO, together with Guinea’s Ministry of Health, MSF and the Norwegian Institute of Public Health, in collaboration with other international partners.

“While these compelling results come too late for those who lost their lives during West Africa's Ebola epidemic, they show that when the next Ebola outbreak hits, we will not be defenceless,” said Dr Marie-Paule Kieny, WHO Assistant Director-General for Health Systems and Innovation, and the lead author of the study.

The manufacturer, Merck, Sharpe & Dohme, has received special designations from the United States Food and Drug Administration and the European Medicines Agency allowing for faster-than-usual regulatory review of the vaccine, once it is submitted, so that it can be licensed as soon as possible.

Since Ebola virus was first identified in 1976, relatively small outbreaks have been reported in Africa. But the 2013–2016 West African Ebola outbreak resulting in more than 11 300 deaths, has highlighted the need for a vaccine.

http://www.who.int/mediacentre/news/releases/2016/ebola-vaccine-results

## NCDs in the African Region

Most adults in WHO’s African region have at least one risk factor for developing a noncommunicable disease (NCD), including heart disease, cancer, type 2 diabetes and chronic obstructive pulmonary disease, according to the *Report on the status of major health risk factors for noncommunicable disease*.

“These are diseases that can be life-threatening as well as debilitating, and they place a significant hardship on the region, robbing people and families of those who otherwise should be enjoying their most productive years,” wrote Dr Matshidiso Moeti, WHO’s Regional Director for Africa in the foreword of the report released in December.

“We must do everything we can to reverse these disturbing trends.”

The report is based on data from WHO STEPwise surveys in 33 countries and on global school-based student health surveys in 19 countries in the Region.

It found that the prevalence of hypertension, or high blood pressure, in the African Region is the highest worldwide, affecting an estimated 46% of adults. In half of the countries in the African Region, at least one in three adults was found to be hypertensive.

The estimated prevalence of people who are overweight ranged from 12% in Madagascar to 60% in the Seychelles, with a median level of 35%, the report showed.

http://www.afro.who.int/en/noncommunicable-diseases/npc-publications.html

**Cover photo**

This month’s cover photo shows migrant workers in India returning home to Nepal to celebrate the festival of Vivah Panchami.

*The* *2030 agenda for sustainable development *recognizes the positive contributions of migrants and their fundamental role in sustainable development. However, many migrants and refugees are vulnerable with limited access to quality health services. Target 8 of SDG 3 calls on countries to achieve universal health coverage, so that no one is left out.

**Figure:**
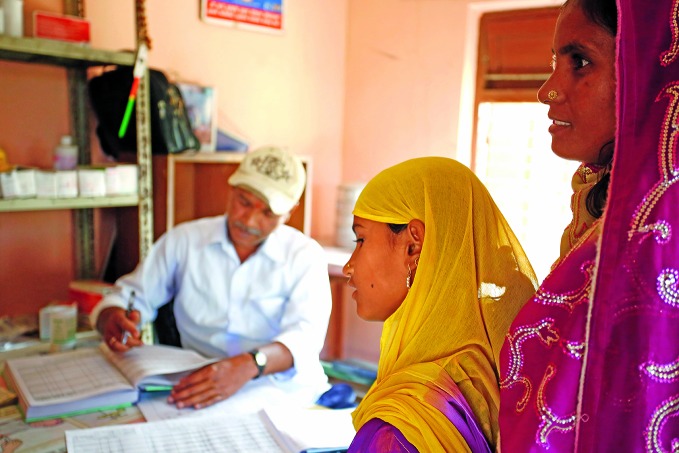
IOM/Sudipto Das

## Guinea-worm eradication efforts

WHO received a request last month from Sudan’s health ministry to evaluate the country’s eligibility for certification as being free of dracunculiasis (guinea-worm disease). 

Sudan was once endemic for the parasitic disease but has reported no indigenous cases for the past three years. 

Meanwhile, last year no cases were reported in Mali, one of the four remaining endemic countries. 

“Mali’s success is an important milestone, but challenges remain for wiping out the disease,” said Dr Dieudonné Sankara, head of the WHO Dracunculiasis Eradication Team, citing Dracunculus medinensis infections in dogs, as well as insecurity and population displacement.

In 2016, the infection was reported to WHO in three other endemic countries: Chad (16 humans, 1013 dogs), Ethiopia (three humans, 14 dogs) and South Sudan (six humans) – a total of 25 cases, three more than in the previous year. 

Efforts to tackle the disease globally started in 1981 when the Interagency Steering Committee for Cooperative Action for the International Drinking Water Supply and Sanitation Decade proposed the elimination of guinea-worm disease as an indicator of the Decade campaign’s success. 

In the same year, the World Health Assembly adopted resolution WHA34.25 recognizing that the Decade campaign presented an opportunity to eliminate guinea-worm disease. This led to WHO and the United States CDC formulating the strategy and guidelines for an eradication campaign. The Carter Center joined the campaign five years later.

To date, WHO has certified 198 countries, territories and areas as dracunculiasis free.

http://www.who.int/dracunculiasis

## The SDGs: more ways to improve health

The 17 sustainable development goals (SDGs) have widened the scope of health on the development agenda, going beyond the millennium development goals by recognizing, in addition, the burden of noncommunicable diseases, mental illness and injuries, and the risks to health during epidemics and humanitarian emergencies.

WHO Director-General Dr Margaret Chan has assembled a new team that will coordinate work across the Organization, aligning technical programmes with regional offices to drive action towards achieving the goals in countries.

Making progress towards these goals requires work across many sectors.

“Less than 4% of the US$ 7 trillion spent on health each year is devoted to the prevention of illness,” said Dr Chris Dye, Director of Strategy, Policy and Information at WHO Headquarters, who will be leading the new team.

“By acting on a wide range of social, economic and environmental determinants of health, in addition to providing better health services, we could avert 40% of premature deaths by 2030, giving extra years of healthy life to people around the world,” he said.

The team will be supported by Dr Shambhu Acharya, Director, Country Cooperation, and by Dr Nata Menabde, Executive Director at the WHO Office at the United Nations, and their teams.

http://www.who.int/topics/sustainable-development-goals

## Report on health, law and rights

Public health laws can play an important role in advancing the right to health and in creating the conditions for people to live healthy lives, according to a new report produced by WHO and its partners.

The report, entitled *Advancing the right to health: the vital role of law* was released last month and provides guidance on the issues that need to be addressed when developing or amending national public health laws. It includes case studies from around the world to illustrate best practices.

The report was produced by WHO along with the International Development Law Organization; the O’Neill Institute for National and Global Health Law at Georgetown University, United States of America; the Sydney Law School, and the University of Sydney in Australia.

www.who.int/entity/healthsystems/topics/health-law/health_law-report

**Looking ahead**

7 April – World Health Day: this year’s theme is depression

22–31 May – 70th World Health Assembly, Geneva, Switzerland

31 May – World No Tobacco Day. This year’s theme is “tobacco: a threat to development”

